# Agonist-Promoted Phosphorylation and Internalization of the Kappa Opioid Receptor in Mouse Brains: Lack of Connection With Conditioned Place Aversion

**DOI:** 10.3389/fphar.2022.835809

**Published:** 2022-05-16

**Authors:** Chongguang Chen, Peng Huang, Kathryn Bland, Mengchu Li, Yan Zhang, Lee-Yuan Liu-Chen

**Affiliations:** ^1^ Center for Substance Abuse Research and Department of Neural Sciences, Temple University Lewis Katz School of Medicine, Philadelphia, PA, United States; ^2^ Department of Medicinal Chemistry, School of Pharmacy, Virginia Commonwealth University, Richmond, VA, United States

**Keywords:** kappa opioid receptor, receptor phosphorylation, receptor internalization, conditioned place aversion, sedation, motor incoordination, analgesic effect, antipruritic effect

## Abstract

Selective kappa opioid receptor (KOR) agonists are promising antipruritic agents and analgesics. However, clinical development of KOR agonists has been limited by side effects, including psychotomimetic effects, dysphoria, and sedation, except for nalfurafine, and recently. CR845 (difelikefalin). Activation of KOR elicits G protein- and β-arrestin-mediated signaling. KOR-induced analgesic and antipruritic effects are mediated by G protein signaling. However, different results have been reported as to whether conditioned place aversion (CPA) induced by KOR agonists is mediated by β-arrestin signaling. In this study, we examined in male mice if there was a connection between agonist-promoted CPA and KOR phosphorylation and internalization, proxies for β-arrestin recruitment *in vivo* using four KOR agonists. Herein, we demonstrated that at doses producing maximal effective analgesic and antiscratch effects, U50,488H, MOM-SalB, and 42B, but not nalfurafine, promoted KOR phosphorylation at T363 and S369 in mouse brains, as detected by immunoblotting with phospho-KOR-specific antibodies. In addition, at doses producing maximal effective analgesic and antiscratch effects, U50,488H, MOM-SalB, and 42B, but not nalfurafine, caused KOR internalization in the ventral tegmental area of a mutant mouse line expressing a fusion protein of KOR conjugated at the C-terminus with tdTomato (KtdT). We have reported previously that the KOR agonists U50,488H and methoxymethyl salvinorin B (MOM-SalB) cause CPA, whereas nalfurafine and 42B do not, at doses effective for analgesic and antiscratch effects. Taken together, these data reveal a lack of connection between agonist-promoted KOR-mediated CPA with agonist-induced KOR phosphorylation and internalization in male mice.

## Introduction

The kappa opioid receptor (KOR) is present in neuronal pathways involved in transmission of pain and itch sensations [reviewed in (Tseng and Hoon, 2022)]. KOR agonists produce analgesic (von Voigtlander et al., 1983) and anti-itch [reviewed in (Inan and Cowan, 2022)] effects; however, they also cause unwanted effects, such as dysphoria, psychotomimetic effects, motor incoordination, and sedation in humans (Pfeiffer et al., 1986; Pande et al., 1996; Walsh et al., 2001; Wadenberg, 2003; MacLean et al., 2013). In rodents, KOR agonists produce analgesia, anti-itch, motor incoordination, sedation, and conditioned place aversion (CPA), an animal model of dysphoria (von Voigtlander et al., 1983; Mucha and Herz, 1985; Simonin et al., 1998). Clinical development of KOR agonists as analgesics and antipruritic agents has been limited by the side effects, except for nalfurafine, and recently, difelikefalin (formerly CR845). Nalfurafine has been approved in Japan for treatment of pruritus associated with chronic kidney or liver, or kidney dialysis [reviewed in (Miyamoto et al., 2022)]. At the therapeutic doses of nalfurafine, dysphoria, hallucination, motor incoordination, and sedation were not reported as major adverse events in humans (Kumagai et al., 2010) [reviewed in (Miyamoto et al., 2022)]. In animals, whether nalfurafine causes side effects depends on the dose used [reviewed in (Zhou et al., 2022)]. It has been demonstrated that nalfurafine produces antipruritic and antinociceptive effects in dose ranges lower than those causing side effects, including conditioned place aversion (CPA), hypolocomotion, motor incoordination, consistent with human data (Tsuji et al., 2001; Liu et al., 2019). However, nalfurafine did cause CPA, motor incoordination, and hypolocomotion at higher doses (Endoh et al., 1999; Dunn et al., 2019) [reviewed in (Zhou et al., 2022)]. Difelikefalin, a peripherally acting KOR peptide agonist, was recently approved by FDA for the treatment of systemic itch in hemodialysis patients (Fishbane et al., 2020) (https://www.accessdata.fda.gov/scripts/cder/daf/index.cfm?event=overview.process&ApplNo=214916).

The KOR belongs to the rhodopsin subfamily of the G protein-coupled receptor (GPCR) superfamily. At the cellular level, activation of the KOR stimulates Gi/o proteins and promotes receptor phosphorylation. Phosphorylated KOR recruits β-arrestins, which leads to desensitization and internalization of the receptor (Liu-Chen, 2004) as well as β-arrestin-mediated signaling (Bruchas and Chavkin, 2010; Brust, 2022; Cahill et al., 2022). Activation of Gi/o proteins inhibits adenylyl cyclases and Ca^++^ channel conductance and enhances activities of K^+^ channels and ERK1/2 (early phase), and β-arrestin-mediated signaling includes activation of ERK1/2 (late phase) and p38 MAPK (Bruchas and Chavkin, 2010; Brust, 2022; Cahill et al., 2022).

Biased agonism or functional selectivity is a popular concept in GPCR pharmacology [reviewed in (Luttrell et al., 2015; Rankovic et al., 2016)]. Per this concept, G protein signaling and β-arrestin signaling produce different *in vivo* behaviors. Therefore, agonists preferentially activating G protein or β-arrestin signaling may have therapeutic advantages with fewer side effects than unbiased agonists. It is generally agreed that KOR agonists produce analgesic and anti-itch effects *via* G protein signaling pathways [reviewed in (Brust, 2022)]. However, literature on the role of β-arrestin signaling in CPA resulting from KOR activation is inconsistent [reviewed in (Brust, 2022)]. Investigations by Roth’s group and Bohn’s group showed that in male mice, β-arrestin2 deletion decreased impaired rotarod performance induced by KOR agonists, but did not affect KOR agonist-produced analgesia, anti-scratch effect, CPA, or hypolocomotion (Morgenweck et al., 2015; White et al., 2015). Chavkin and coworkers demonstrated that GRK3 deletion in mice abolished U50,488H-induced CPA (Bruchas et al., 2007), and KOR-promoted CPA was GRK3 and p38 MAPK dependent (Bruchas et al., 2007; Bruchas et al., 2011; Ehrich et al., 2015). Both GRK3 and β-arrestin2 interact with many GPCRs; thus, the possibility that the impact of either deletion may be through indirect effects on other GPCRs cannot be excluded. In addition, β-arrestin1 may compensate for the absence of β-arrestin2, thus, obscuring the role of β-arrestin2. In this study, we examined KOR agonists for their activities in promoting KOR phosphorylation in wild-type mice to circumvent these issues and determined whether there was a relationship between KOR-mediated behaviors and KOR phosphorylation.

Agonist-induced KOR phosphorylation and internalization in brains may be used as proxies for β-arrestin recruitment. We have previously demonstrated the selective KOR agonist U50,488H-induced phosphorylation of the mouse KOR expressed in cultured cells at S356, T357, T363, and S369 (Chen et al., 2016). We generated and purified antibodies against the mouse KOR peptides containing pS356/pT357, pT363, or pS369 and found that antibodies were specific for phosphorylated forms of KOR in cells (Chen et al., 2016). We further characterized pT363 and pS369 antibodies using brains of wild-type and KOR knockout mice treated with vehicle or U50,488H. We found that the antibodies were specific for KOR phosphorylated at pT363 and pS369 in brains and that U50,488H promoted KOR phosphorylation in a dose-dependent manner (Liu et al., 2020; Chen et al., 2022; Huang et al., 2022).

We previously generated a mutant mouse line expressing a fusion protein of KOR conjugated with the fluorescent protein tdTomato 5′ to the stop codon (KtdT). U50,488H caused internalization of KOR-tdT in the VTA of KtdT mice (Chen et al., 2020).

In this study, we examined whether there was a connection between KOR agonist-induced KOR phosphorylation in the mouse brain and internalization in the mouse ventral tegmental area (VTA) and KOR agonist-produced side effects, such as CPA and hypolocomotion. Four KOR agonists [U50,488H, methoxymethyl salvinorin B (MOM-SalB), nalfurafine, and 42b] were included in the study. U50,488H is the first nonpeptide selective KOR agonist and has the arylacetamide basic structure (von Voigtlander et al., 1983). MOM-SalB is a longer-acting analog of salvinorin A (Wang et al., 2008), which is a highly selective and the first non-nitrogenous KOR agonist (Roth et al., 2002). Nalfurafine is a moderately selective KOR agonist and has 4,5-epoxymorphinan structure (Seki et al., 1999). 42b is 3-deoxy nalfurafine and has moderate selectivity for the KOR (Nagase and Fujii, 2013; Cao et al., 2020).

We previously performed *in vitro* and *in vivo* pharmacological characterization of these compounds (Wang et al., 2008; Liu et al., 2019; Cao et al., 2020). EC_50_ values and maximal effects of the four KOR agonists in stimulating KOR-mediated [^35^S]GTPγS binding are shown in Supplementary Table S1. In addition, EC_50_ values and maximal effects of nalfurafine and 42b in stimulating [^35^S]GTPγS binding mediated by mu, delta, and nociceptin/orphanin FQ receptors (MOR, DOR, and NOR, respectively) are included in Supplementary Table S1 (Wang et al., 2008; Liu et al., 2019; Cao et al., 2020). The four agonists dose-dependently produced analgesic effects in the formalin test and inhibition of scratching induced by compound 48/80 (Supplementary Figures S1 and S2) (Liu et al., 2019; Cao et al., 2020). We have also demonstrated that s.c. administration of U50,488H (0.25–10 mg/kg) and MOM-SalB (0.01–0.3 mg/kg) caused CPA, whereas nalfurafine (2.5–20 μg/kg) and 42B (1–5 mg/kg) did not (Liu et al., 2019; Cao et al., 2020) (Supplementary Figure S3). In addition, U50,488H (5 mg/kg) and 42B (5 mg/kg), but not nalfurafine (20 μg/kg), induced hypolocomotion (Liu et al., 2019; Cao et al., 2020) (Supplementary Figure S4). Moreover, U50,488H (5 mg/kg) and 42B (3, 5 mg/kg) profoundly impaired rotarod performance, but nalfurafine (20 μg/kg) had slight effects (Supplementary Figure S5). These data are included in the Supplementary Material to enhance readability. In additional experiments, we demonstrated that MOM-SalB (200 μg/kg) greatly inhibited novelty-induced hyperlocomotion and impaired rotarod performance, which are presented in Supplementary Figures S4 and S5.

## Materials and Methods

### Animals

Adult male CD-1 mice (Charles River Laboratories, Wilmington, MA, USA), 30–35 g, were used for most experiments. Adult mice expressing a fusion protein of the KOR conjugated with the fluorescent protein tdTomato (tdT) (KtdT) in C57BL/6N background (25–30 g) (Chen et al., 2020) were used in agonist-promoted KOR internalization experiments. Mice were maintained in 12/12 light–dark cycle with *ad libitum* access to food and water. Experiments were conducted in accordance with the National Institutes of Health Guide for the Care and Use of Laboratory Animals. All methods used were preapproved by the Institutional Animal Care and Use Committee at the Temple University.

### Drugs

(±) U50,488H and nalfurafine were obtained from the National Institute on Drug Abuse (Bethesda, MD, United States). 42B was prepared in-house following the method of Nagase and Fujii (2013). MOM-SalB was provided by Dr. David Y. Lee of McLean Hospital (Belmont, MA, United States). The vehicle for U50,488H, nalfurafine, and 42B was saline, while that for MOM-Sal B was Kolliphor:ethanol:water (1:1:98) (KEW). Unless otherwise noted, KOR agonists and vehicles were injected subcutaneously (s.c.) in a volume of 0.1 ml/10 g body weight.

### Materials

The following reagents were obtained from indicated companies: protease inhibitor tablets and SuperSignal West Pico chemiluminescence reagent, Pierce/Thermo Fisher Scientific (Waltham, MA, United States); dodecyl-β-D-maltoside (DDM), Pansorbin and PVDF membranes, EMD Millipore (Billerica, MA, United States); normal goat serum, VectaShield mounting media, Vector Labs (Burlingame, CA, United States). The following materials were purchased from Sigma-Aldrich (St. Louis, MO, United States): paraformaldehyde (PFA), compound 48/80, Kolliphor EL (formerly known as Cremophor EL), IPGEL (previously named NP-40), sodium deoxycholate, Na_4_ pyrophosphate, Na_2_ glycerophosphate, NaF, Na orthovanadate, dithiothreitol (DTT), sodium dodecyl sulfate (SDS), and Triton X-100. Other commonly used chemicals were obtained from Sigma-Aldrich or ThermoFisher Scientific.

### Antibodies

Rabbit antibodies against KOR (371–380) peptide [anti-KC (PA847)], pT363-KOR, and pS369-KOR were generated, purified, and characterized as we described (Chen et al., 2016). In addition, guinea pig antibodies [anti-KC (PA5699)] were custom-generated against KOR (371–380)-keyhole limpet hemocyanin (KLH) by Covance Co. (Princeton, NJ, United States) and similarly purified and characterized. Peptides and phospho-peptides used in antibody generation and purification were custom synthesized by EZBiolab (Carmel, IN, United States). We have shown that in immunoblotting, pT363-KOR and pS-369-KOR antibodies are specific for phosphorylated KOR in cultured cells and in mouse brains (Chen et al., 2016; Liu et al., 2020; Chen et al., 2022; Huang et al., 2022).

Rabbit anti-red fluorescent protein (RFP) antibody was purchased from Rockland (catalog no. 600-401-379, Limerick, PA, United States). Mouse monoclonal antibodies against tyrosine hydroxylase (anti-TH) were from ImmunoStar (catalog #22941, Hudson, WI, United States). Mouse monoclonal antibody against S6 was from Santa Cruz (SC-74459, Santa Cruz, CA, United States). Goat anti-rabbit IgGs conjugated with Alexa 594 (A11012) and goat anti-mouse IgGs conjugated with Alexa 488 (A11001) were from ThermoFisher (Waltham, MA, United States). Horseradish peroxidase (HRP)-conjugated mouse anti-rabbit IgG light chain was obtained from Jackson ImmunoResearch Labs, Inc. (West Grove, PA, United States).

### Agonist-Promoted Kappa Opioid Receptor Phosphorylation in Mouse Brains

The experiments were performed per our published procedures (Liu et al., 2020; Huang et al., 2022). Male adult CD-1 mice were injected s. c with vehicle, U50,488H (5 mg/kg), 42B (5 mg/kg), MOM-Sal B (200 μg/kg, s.c.) or nalfurafine (30 μg/kg, s.c.), and 30 min later, animals were euthanized. Brains were removed immediately, frozen, and stored in −80°C. The dose used was within the effective dose ranges in the formalin test and antiscratch test, which produced maximal responses in both tests, and at 30 min, effects of the drugs were fully manifested.

All the following procedures were conducted at 4°C unless indicated otherwise, and a rotator mixer was used for incubation, mixing, and washing. Four mouse brains were pooled as one sample, due to the low KOR expression level, and homogenized and solubilized with 1% IPGEL (NP-40), 1% sodium deoxycholate, 0.1% sodium dodecyl sulfate (SDS) in 25 mM Tris-Tris HCl buffer, pH 7.4, containing phosphatase inhibitors, protease inhibitors, and Pansorbin. Proteins interacting with Pansorbin were removed by centrifugation. KOR in the supernatant was immunoprecipitated twice in tandem. The first immunoprecipitation was performed with rabbit anti-KC (PA847) and Pansorbin, centrifuged, and the pellet washed. Following dissociation of immunoprecipitated proteins from Pansorbin and removal of Pansorbin, the second immunoprecipitation was done with guinea pig anti-KC (PA5699) and Pansorbin, centrifuged, and the pellet washed. KOR was eluted from KOR Ab-Pansorbin complex with 0.1 mg/ml of KOR (371–380) peptide (the peptide antigen, amino acid sequence: RDVGGMNKPV) at room temperature. The combined eluate was concentrated to 20 µl using an Amicon Concentrator with a 50-kDa cutoff. The eluate was mixed with loading buffer, heated, and subjected to SDS-PAGE (precast gel 4%–12%, Invitrogen). Immunoblotting was performed with rabbit anti-pT363 or anti-pS369 (1 μg/ml) followed by HRP-conjugated mouse anti-rabbit IgG light chain and reaction with enhanced chemiluminescence reagents. Images were captured using LAS 1000 plus camera (Fuji Photo Film Co.) and quantified using the ImageGauge software (Version 4.1). The blot was stripped and re-blotted with rabbit anti-KC (PA847) for total KOR. For quantitation, p-KOR staining intensity was normalized against that of the total KOR in the same lane. The resulting data were then normalized against those of U50,488H.

### Agonist-Induced Kappa Opioid Receptor Internalization in the Ventral Tegmental Area of Homozygous KtdT Mice

The experiments were performed per our published procedures (Chen et al., 2020). Generation, genotyping, and characterization of KtdT mice were described previously (Chen et al., 2020). Following habituation to handling and injection (s.c.) every day for 3 days, adult male homozygous KtdT (KOR^tdT/tdT^) (KtdT/KtdT) mice in C57BL6/N background were injected with saline, KEW vehicle, U50,488H (5 mg/kg), nalfurafine (30 μg/kg), 42B (5 mg/kg), or MOM-SalB (200 μg/kg). Thirty minutes later, mice were anesthetized, transcardially perfused with 4% paraformaldehyde (PFA). The brains were removed, postfixed overnight, and then placed in 30% sucrose for up to 72 h for cryoprotection. Brains were frozen and cut at −18°C with cryostat (Leica CM3050S) to obtain coronal sections (30 μm) containing the ventral tegmental area (VTA), which were placed in 1× PBS (8.2 mM Na_2_HPO_4_, 1.8 mM KH_2_PO_4_, NaCl 134 mM, KCl 2.7 mM, pH7.5) plus 0.05% NaN_3_ for short-term storage. IHC was performed with anti-RFP and tyrosine hydroxylase (TH) antibody for examining colocalization of KtdT with TH. Floating sections were rinsed and incubated with the blocking buffer (5% normal goat serum, 0.1 M glycine, 0.3% Triton X-100 in 1× PBS). IHC was performed with rabbit anti-RFP (1/1,000) and mouse anti-tyrosine hydroxylase monoclonal antibody (1:1,000), respectively, in the staining buffer (3% BSA, 0.3% Triton X-100 in 1× PBS) at 4°C overnight and washed. Sections were then incubated with AlexaFluor594-conjugated goat anti-rabbit IgG (1:1,000) for tdT and goat anti-mouse IgG conjugated with AlexaFluor 488 (1:1,000) for TH overnight at 4°C. After washes, sections were mounted on fluorescence-free glass slides with Vectashield containing DAPI and placed at 4°C for storage for up to 2 months. Sections were examined under a fluorescence microscope (Nikon, ECLIPSE TE300), and some were further examined under a confocal microscope (Nikon A1R).

### Quantitation of Kappa Opioid Receptor Internalization

Quantitation was performed as we described previously (Chen et al., 2020). IHC was performed with rabbit anti-RFP (1/1,000) and mouse monoclonal antibody against the ribosomal protein S6 (S6) (1/500), a cytosolic protein, followed by AlexaFluor594-conjugated goat anti-rabbit IgG (1:1,000) for tdT and goat anti-mouse IgG conjugated with Alexa 488 (1:1,000) for S6 overnight at 4°C. S6 is used to define cytosol of KOR-expressing neurons for quantitation purpose as S6 is present in every cell, but TH is not, and some cells express KOR, but not TH and *vice versa* (Chen et al., 2020). Measurement and calculation of KOR translocation was performed with ImageJ (Fiji version). The nuclear region of interest (ROI) was defined by DAPI (circle 1). Intracellular ROI was defined by drawing a circle with a single pixel line around the perimeter of the S6 staining (circle 2). The circle 2 was uniformly enlarged by three pixels to define total cell area (circle 3). The ROIs were drawn on three focal planes across the Z-stack of each neuron at intervals ≥ five focal planes and registered to ROI Manager of Fiji. Background autofluorescence was corrected by the Rolling Ball (100-μm radius) algorithm of Fiji. The intensities of KtdT fluorescence were measured for each ROI. Total receptor was defined by circle 3 minus circle 1 and cell surface receptor as circle 3 minus circle 2. The fluorescence intensity was normalized by dividing the intensity by the respective area. The results of three focal planes/neuron were averaged and counted as the value of one neuron. Forty to 50 neurons/mouse were measured, three mice/treatment, and the mean values were used for statistical analysis. Image quantitation analyses were done by an observer blinded to the treatment group.

## Results

### Doses of U50,488H, Methoxymethyl Salvinorin B, Nalfurafine, and 42B Used in Kappa Opioid Receptor Phosphorylation and Internalization

We previously showed that U50,488H, MOM-SalB, nalfurafine, and 42B produced antinociceptive effects in the second phase of the formalin test with A_50_ values of 0.58 mg/kg, 17.0 μg/kg, 5.8 μg/kg, and 2.08 mg/kg, respectively (Liu et al., 2019; Cao et al., 2020) (Supplementary Figure S1). In addition, U50,488H, MOM-SalB, nalfurafine, and 42B inhibited compound 48/80-induced scratching, and their A_50_ values were determined to be 2.07 mg/kg, 70.2 μg/kg, 8.0 μg/kg, and 2.95 mg/kg, respectively (Liu et al., 2019; Cao et al., 2020) (Supplementary Figure S2).

The doses of U50,488H, MOM-SalB, nalfurafine, and 42b used in the KOR phosphorylation and internalization experiments were 5 mg/kg, 200 μg/kg, 30 μg/kg, and 5 mg/kg, respectively. The doses are in the dose ranges that produce effective analgesic and antiscratch effects and cause maximal effects in the formalin test and the anti-scratch test (Supplementary Figures S1 and S2). These doses are within the dose ranges used in the experiments of CPA, rotarod test, and inhibition of novelty-induced hyperlocomotion (Supplementary Figures S3–S5), except for nalfurafine. We used 30 μg/kg of nalfurafine instead of 20 μg/kg because in the preliminary experiments, 20 μg/kg did not cause KOR phosphorylation, and we used a higher dose to confirm the finding.

### U50, 488H, Methoxymethyl Salvinorin B, and 42B Caused Significant Kappa Opioid Receptor Phosphorylation, but Nalfurafine Did Not

We have demonstrated previously that T363 and S369 in the KOR are the primary phosphorylation sites, and phosphorylation of T363 and S369 is required for S356/T357 phosphorylation (Chen et al., 2016). Therefore, our phosphorylation studies were focused on T363 and S369. We have shown that in immunoblotting, pT363-KOR and pS-369-KOR antibodies are specific for phosphorylated KOR in cultured cells (Chen et al., 2016) and in mouse brains (Liu et al., 2020; Chen et al., 2022; Huang et al., 2022).

**FIGURE 1:**
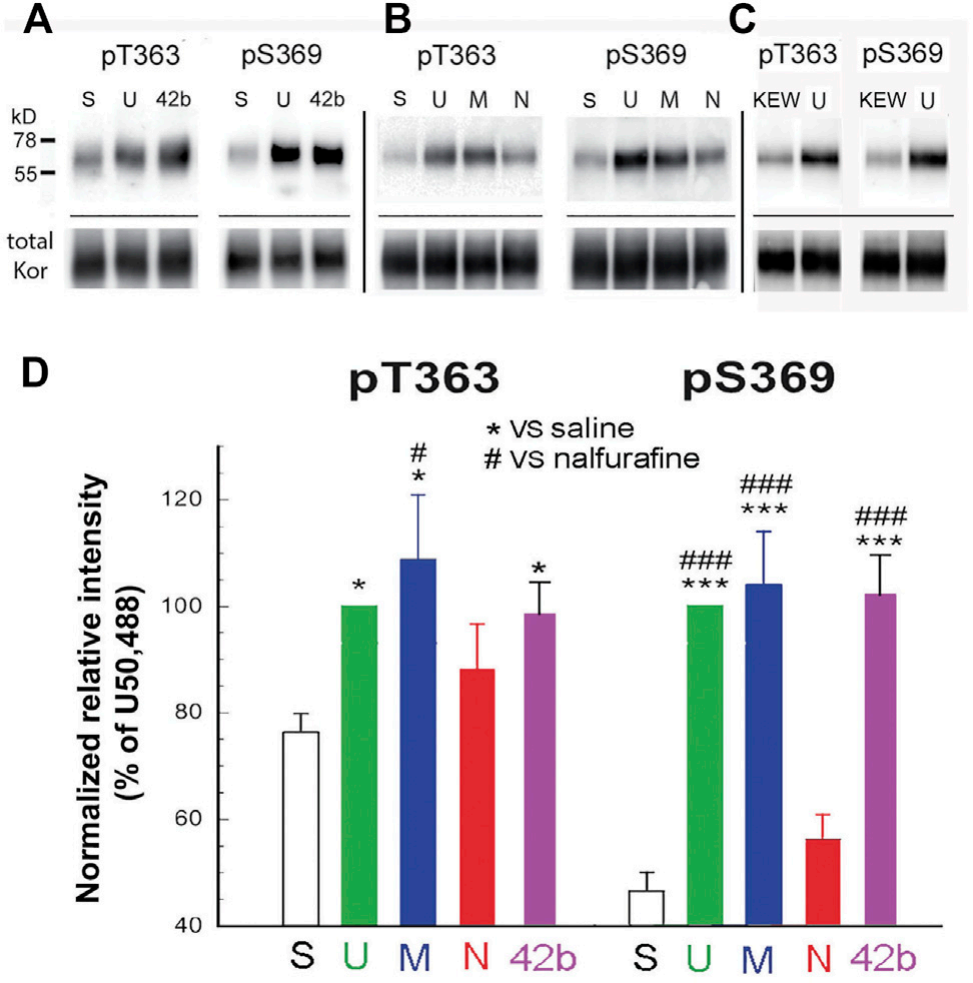
U50,488H, MOM-SalB, and 42b, but not nalfurafine, promoted robust kappa opioid receptor (KOR) phosphorylation in mouse brains. Male CD-1 mice were injected (s.c.) with saline, Kolliphor EL: ethanol: water (1:1:98) (KEW), U50,488H (5 mg/kg), MOM-SalB (200 μg/kg), nalfurafine (30 μg/kg) or 42b (5 mg/kg) and euthanized 30 min later. Four brains were pooled as one sample because of low levels of KOR. KOR was partially purified by immunoprecipitation, resolved with SDS-PAGE and IB was performed with rabbit anti-pT363 and anti-pS369 antibodies. The blot was then stripped and re-blotted for total KOR with purified rabbit antibodies against the KOR (371–380) peptide. **(A–C)** A representative blot. S, saline; U, U50,488H; M, MOM-SalB; N, nalfurafine; 42b; KEW, vehicle for MOM-SalB. For each experiment, vehicle- and U50,488H-treated samples were included. **(C)** KEW did not increase KOR phosphorylation, compared with saline. Because of this, we performed KEW on only one sample of brains pooled from four mice. Thus, the KEW data were not included in **(D)**. **(D)** Quantitation of agonist-promoted KOR phosphorylation. For each lane, P-KOR staining intensity was normalized against that of the total KOR. The resulting data were then normalized against that of U50,488H. Each value is mean ± SEM (*n* = 3). Data were analyzed by one-way ANOVA followed by *post-hoc* Tukey multiple comparisons Test. For pS363, *p < 0.05, U or M vs. S; ^#^ *p* < 0.05, M vs. N. For, pS369, ***p < 0.001, U, M or 42b vs. S; ^###^ *p* < 0.001, U, M or 42b vs. N.

Mice were injected (s.c.) with U50,488H (5 mg/kg), MOM-SalB (200 μg/kg), nalfurafine (30 μg/kg), or 42B (5 mg/kg), killed 30 min later, and brains were removed. The doses used were identical to the highest doses for the rotarod and locomotor activity tests, except nalfurafine, which was higher (30 vs. 20 μg/kg). Following partial purification of KOR in the brain by immunoprecipitation, KOR phosphorylation at T363 and S369 was detected by immunoblotting with phospho-specific antibodies. U50,488H, MOM-SalB, and 42B induced significant KOR phosphorylation at T363, whereas nalfurafine, even at 30 μg/kg, showed a trend of increase, but did not reach statistical significance. In addition, U50,488H, MOM-SalB, and 42B caused profound increases in KOR phosphorylation at S369, but nalfurafine did not (Figure 1).

### U50, 488H, Methoxymethyl Salvinorin B, and 42B Induced Kappa Opioid Receptor Internalization in the Ventral Tegmental Area, but Nalfurafine Did Not

**FIGURE 2:**
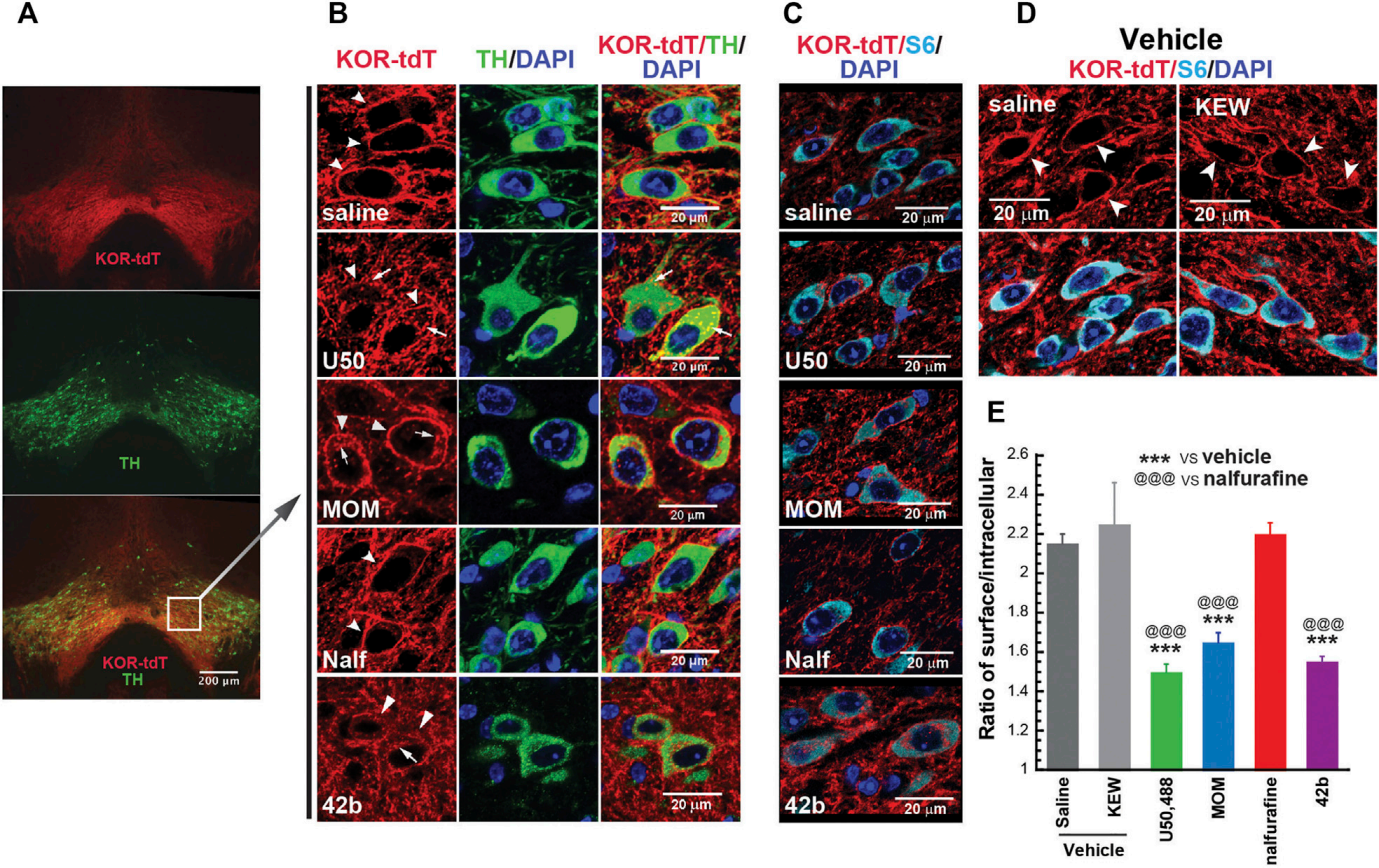
Differential abilities of KOR agonists in promoting KOR internalization in the ventral tegmental areas (VTAs) of male KtdT/KtdT mice. Male KtdT/KtdT mice were injected with saline, Kolliphor EL:ethanol:water (1:1:98) (KEW, vehicle for MOM-SalB), U50,488H (5 mg/kg), MOM-SalB (200 μg/kg), nalfurafine (30 μg/kg), or 42b (5 mg/kg), and 30 min later perfused transcardially with 4% paraformaldehyde. **(A,B)** KOR-tdT and tyrosine hydroxylase (TH) colocalized in some VTA neurons, and treatment with U50,488, MOM-SalB, and 42b induced KOR internalization VTA, but nalfurafine did not. Coronal sections of frozen brains were obtained and processed for double immunohistochemistry (IHC) with rabbit antibodies against rabbit anti-red fluorescent protein (RFP) (red) and mouse antibodies against tyrosine hydroxylase (TH) (green). Sections were also stained with DAPI for nuclei (blue). **(A)** Epifluorescence microscopy images of the VTA. Scale bar = 200 μm. **(B)** A layer of confocal microscopy image. Scale bar = 20 μm. Note that many neurons express both TH and KtdT. Arrows point to internalized receptors, whereas arrow heads indicate receptors on membranes. The experiment was performed on three mice for each group **(C–E)** Quantitation of agonist-induced KOR internalization. **(C)** IHC was performed on coronal sections containing the VTA with antibodies against tdT (red) and S6 (cyan), a ribosomal protein in cytosol for identification of all the cells. Sections were also stained with DAPI for nuclei (blue). **(D)** KEW did not cause KOR internalization, like saline. KEW is the vehicle we used for MOM-SalB. **(E)** Quantitation of KOR internalization was performed on three sections/mouse at different rostral–caudal positions. Fifteen to 20 neurons per section and three focal planes per neuron from image stacks were measured. Thus, on the average, about 50 neurons/mouse were quantified for internalization as described in the *Methods* section. Experiments were performed on three mice/group. ***p < 0.001, compared with saline; ^@@@^ *p* < 0.001, compared with nalfurafine, by one-way ANOVA followed by Tukey’s multiple comparisons test. Each value is mean ± SEM (*n* = 3 mice).

Agonist-promoted KOR internalization was used as a proxy of β-arrestin recruitment. We examined if U50,488H, MOM-SalB, nalfurafine, and 42B promoted KOR internalization in the VTA of KtdT/KtdT mice. As shown in Figures 2A,B, many neurons in the VTA are stained positive for both tyrosine hydroxylase (TH) and KtdT, indicating colocalization of TH and KOR in these cells. In sections of vehicle-treated mice, KOR-tdT immunoreactivities clearly are present on plasma membranes as continuous lines encircling cells (Figures 2B–D). In the U50,488H-treated group, the line of KOR-tdT immunoreactivity was broken, and the immunoreactivity inside the cells was increased. When overlapped with TH immunoreactivity, intracellular KOR-tdT immunoreactivity appeared as yellow or orange dots in cytoplasmic space, indicating internalization of KtdT (Figure 2B). Similarly, MOM-SalB or 42b treatment promoted KtdT internalization in the VTA (Figure 2B). In contrast, in the nalfurafine-treated group, KtdT immunoreactivity was clearly on plasma membranes, and little KtdT was present in cytoplasmic space (Figure 2B).

For quantitation of KOR internalization, IHC was performed on sections containing the VTA with antibodies against RFP (red) and the cytosolic protein S6 (cyan) because S6 is present in every neuron, but TH is not (Figure 2C). S6 IHC staining facilitated visualization of intracellular KOR-tdT. Figure 2D shows that KEW, the vehicle for MOM-SalB, did not cause KOR internalization, like saline. KOR-tdT internalization was calculated as the ratio of cell surface KtdT/intracellular KtdT (Chen et al., 2020). Figure 2E shows that U50,488H, MOM-SalB, and 42b, but not nalfurafine, decreased the ratio of cell surface KtdT/intracellular KtdT, indicating that U50,488H, MOM-SalB, and 42b, but not nalfurafine, promotes KOR internalization in neurons in the VTA.

## Discussion

Table 1 summarizes the activities of the four KOR agonists at indicated doses to produce analgesia, antiscratch effect, CPA, motor incoordination, and hypolocomotion in male mice as well as their abilities to promote KOR phosphorylation in male mouse brains and KOR internalization in VTA neurons of male KtdT mice. U50,488H and MOM-SalB produced all the effects, whereas 42B did not cause CPA, but caused all the other effects. Nalfurafine produced analgesic and antiscratch effects and slightly impaired rotarod performance, but did not produce CPA, hypolocomotion, KOR phosphorylation, or internalization. 42B and nalfurafine did not produce CPA, yet 42b, not nalfurafine, promoted profound KOR phosphorylation and internalization, suggesting that there is no overall connection between KOR phosphorylation/internalization and CPA. Because all the experiments were performed in male mice, this conclusion applies to male mice only (see discussion on female mice in the CPA section below).

**TABLE 1:** Summary of effects of the four kappa opioid receptor (KOR) agonists on behaviors and KOR phosphorylation and internalization.

	U50,488H	MOM-SalB	Nalfurafine	42B
Analgesia (formalin test)	Yes A50 = 0.58 mg/kg	Yes A50 = 17.0 μg/kg	Yes A50 = 5.8 μg/kg	Yes A50 = 2.08 mg/kg
Anti-scratch (compound 48/80)	Yes A50 = 2.07 mg/kg	Yes A50 = 70.2 μg/kg	Yes A50 = 8.0 μg/kg	Yes A50 = 2.95 mg/kg
CPA	Yes (0.25–10 mg/kg)	Yes (10–300 μg/kg)	No (2.5–20 μg/kg)	No (1–5 mg/kg)
Rotarod: motor incoordination	Yes (2, 5 mg/kg)	Yes (70, 200 μg/kg)	Slight effect (20 μg/kg)	Yes (1, 3, 5 mg/kg)
Hypolocomotion	Yes (5 mg/kg)	Yes (200 μg/kg)	No (20 μg/kg)	Yes (5 mg/kg)
KOR phosphorylation	Yes (5 mg/kg)	Yes (200 μg/kg)	No (30 μg/kg)	Yes (5 mg/kg)
KOR internalization	Yes (5 mg/kg)	Yes (300 μg/kg)	No (30 μg/kg)	Yes (5 mg/kg)

As agonist-promoted KOR phosphorylation and internalization were performed with brains of mice injected with vehicle or KOR agonists, it is reasonable to make such connection because pharmacokinetic characteristics, such as absorption, metabolism, and blood brain penetration, are identical for the same drug in behavior and biochemical experiments.

### The Abilities of Agonists to Promote Kappa Opioid Receptor Phosphorylation are Related Their Abilities to Cause Kappa Opioid Receptor Internalization in the Ventral Tegmental Area in Male Mice

Among the four KOR agonists, U50,488H, MOM-SalB, and 42b, but not nalfurafine, promoted KOR phosphorylation in the mouse brain and KOR internalization in the mouse VTA. Thus, there appears to be a relationship between KOR phosphorylation in mouse brains and KOR internalization in the VTA. We were not able to conduct the KOR phosphorylation experiments using the VTA because the number of VTAs required made it prohibitive. Instead, agonist-induced KOR phosphorylation was conducted using whole brains (minus the cerebellum), and four brains were pooled as one sample because of the very low level of KOR in the mouse brains. Immunoprecipitation of KOR was performed to enrich KOR before immunoblotting for phosphorylated and total KOR. Whether KOR phosphorylation in the whole mouse brain reflects that in the VTA is not known.

### Conditioned Place Aversion is Not Related to Kappa Opioid Receptor Phosphorylation and Internalization in Male Mice

Here we demonstrated using male mice that at doses producing maximal antiscratch and analgesic responses, U50,488H, MOM, and 42b, but not nalfurafine, promoted KOR phosphorylation at T363 and S369 in mouse brains and induced KOR internalization in VTA neurons of KtdT/KtdT mice. Our data showed male mice at doses effective in the analgesia and antiscratch tests, while U50,488H, and MOM caused CPA, nalfurafine, and 42b did not (Liu et al., 2019; Cao et al., 2020). Thus, the finding that 42b promoted robust KOR phosphorylation and internalization, but did not cause CPA in male mice, indicates that KOR phosphorylation and internalization are not related to KOR-mediated CPA in male mice.

We have recently generated a mouse line expressing a KOR mutant with the four phosphorylation sites substituted with alanines (K4A). K4A mutations eliminated U50,488H-promoted KOR phosphorylation, but did not affect U50,488H (2 or 5 mg/kg)-induced CPA in male mice (Huang et al., 2022). The observation in the current study (conducted in male mice) that KOR phosphorylation is not related to CPA is consistent with those in male K4A mice. However, in female mice, K4A mutations abolished U50,488H (2 mg/kg)-induced CPA. These results indicate that there are sex differences in the role of KOR phosphorylation in CPA, which will require further studies in the future.

Our results are consistent with those of White et al. (2015) that β-arrestin2 deletion in mice did not affect U69,593-induced CPA. In contrast, Chavkin and others reported that KOR agonist-induced CPA was mediated by β-arrestin-dependent p38 MAP activation [reviewed in (Bruchas and Chavkin, 2010)].

In our phosphoproteomics study on mouse brains, we found that U50,488H activated the mTOR pathway in the striatum and cortex, but nalfurafine did not, and inhibition of mTOR with rapamycin blocked U50,488H- and MOM-SalB-induced CPA, indicating that the mTOR pathway is involved in U50,488H- and MOM-SalB-induced CPA (Liu et al., 2019). A logical step would be to examine if the four agonists have differential abilities to activate the mTOR pathway. We previously examined if U50,488H promoted p70S6K phosphorylation in the striatum by immunoblotting with rabbit monoclonal antibodies against phospho-T389 p70S6K and total p70S6K antibodies (#97596 and #9202, respectively, from Cell Signaling Technology, Danvers, MA). We found that the Mr of phospho-T389 p70S6K was ∼85 kDa, presumably p85S6K, whereas that of total p70S6K was ∼70 kDa (Liu et al., 2019), making it difficult to interpret the data.

### Kappa Opioid Receptor Agonist-Induced Hypolocomotion vs. Kappa Opioid Receptor Phosphorylation and Internalization

U50,488H, MOM-SalB, and 42b caused inhibition of novelty-induced hyperlocomotion, whereas nalfurafine did not. Thus, among these four agonists, there appears to be a connection between the abilities of the agonists to cause hypolocomotion and their abilities to promote KOR phosphorylation and internalization.

It should be noted that this connection is not causal relationship. In both males and females, K4A substitutions abolished U50,488H-induced KOR phosphorylation, but did not affect U50,488H-induced hypolocomotion, indicating that KOR phosphorylation does not cause hypolocomotion (Huang et al., 2022). In addition, β-arrestin2 deletion in mice did not affect U69,593-induced hypolocomotion (White et al., 2015).

### Anti-Scratch and Analgesic Effects of KOR Agonists Are Un-Related to Kappa Opioid Receptor Phosphorylation and Internalization

Nalfurafine produced robust antiscratch and analgesic effects (Liu et al., 2019; Cao et al., 2020), but did not promote KOR phosphorylation or internalization. Therefore, there is no relationship between KOR antiscratch and analgesic effects and KOR phosphorylation or internalization. These findings are consistent with those of White et al. (2015) and Morgenweck et al. (2015) that β-arrestin2 deletion in mice had no effect on U69,593-induced analgesia in the hot plate test or the inhibitory effect of chloroquine-promoted scratch by U50,488H. U50,488H produced a similar inhibition of compound 48/80-induced scratching in wild-type mice and mutant mice expressing a phosphorylation-deficient KOR mutant (Huang et al., 2022), also indicating that KOR agonist-promoted antiscratch effects are not related to KOR phosphorylation. The finding that KOR-mediated analgesia is unrelated to KOR phosphorylation and internalization is consistent with the notion that KOR-mediated analgesia is mediated by G protein-signaling pathway.

### Selectivity of the Agonists for the Kappa Opioid Receptor

Among the four agonists, U50,488H is a highly selective KOR agonist (von Voigtlander et al., 1983). MOM-SalB, a longer-acting analog of salvinorin A, shows high selectivity for the KOR (Roth et al., 2002; Wang et al., 2008). Nalfurafine and 42b, in addition to being KOR full agonists, also have activities on other opioid receptors, with 42b having lower KOR/MOR or KOR/DOR selectivity (Supplementary Table S1). In [^35^S]GTPγS binding assay using membranes of cultured cells, 42b is a full agonist at the KOR and DOR and a partial agonist at the MOR, and its selectivity factors for the KOR over MOR and DOR are 8.4 and 13.0, respectively (Supplementary Table S1). We demonstrated that the antiscratch effect of 42b was blocked by 24-h pretreatment with the KOR-selective antagonist norbinaltorphimine, indicating KOR-mediated effects (Cao et al., 2020). Nonetheless, the possibility that 42b may act on the DOR and, to a less extent, the MOR to counteract the aversive effect of KOR activation cannot be entirely excluded. In mice, activation of MOR or DOR causes hyperlocomotion (Michael-Titus et al., 1989; Mickley et al., 1990), whereas stimulation of KOR produces hypolocomotion. At the doses we used, 42b caused profound inhibition of locomotor activity, suggesting that it preferentially acts on the KOR.

### Agonist Biases of the Four Agonists at the Kappa Opioid Receptor

Agonist biases of KOR have been determined using, most commonly, cultured cells or, occasionally, primary neurons (Schmid et al., 2013; Zhou et al., 2013; White et al., 2014; DiMattio et al., 2015; Schattauer et al., 2017; Dunn et al., 2019; Kaski et al., 2019; Liu et al., 2019; Cao et al., 2020). Dose–response curves of KOR agonists in stimulating G protein signaling and in activating β-arrestin signaling are obtained, and in some studies, agonist biases are determined using a reference compound as the unbiased agonist, such as U50,488H or dynorphin A (1–17). The end points for G protein signaling include [^35^S]GTPγS binding, inhibition of forskolin-stimulated adenylyl cyclase, and ERK1/2 phosphorylation. The functional measures of β-arrestin signaling include β-arrestin recruitment using different assays, receptor internalization, and p38 MAPK phosphorylation. The most used methods for quantifying ligand bias use the Black and Leff operational model to calculate transduction coefficients (Black and Leff, 1983; Griffin et al., 2007; Kenakin and Christopoulos, 2013). U50,488H is the prototypic selective KOR agonist (von Voigtlander et al., 1983) and has been considered as an unbiased agonist because it produces the full spectrum of KOR-mediated behaviors. When dynorphin A was used as the reference unbiased compound, U50,488H was determined to be internalization (β-arrestin) biased at the mouse KOR and unbiased at the human KOR (DiMattio et al., 2015). Agonist activities of nalfurafine at the KOR has been found to be G protein biased (Schattauer et al., 2017; Kaski et al., 2019; Cao et al., 2020), unbiased (Liu et al., 2019), or β-arrestin biased (Dunn et al., 2019). At the KOR, 42b is a weakly biased agonist for G protein signaling with U50,488H as the unbiased agonist (Cao et al., 2020). MOM-SalB is not significantly biased at the mouse KOR using dynorphin A as the reference unbiased compound (DiMattio et al., 2015). Determination of agonist biases may be influenced by many factors in the experiments, including the cell line, end point, assay method for the same end point, receptor expression level, and the reference balanced agonist. There are also many factors affecting translation of *in vitro* agonist biases to *in vivo* behavioral effects, including absorption, metabolism, and penetration into the brain of the compound.

### Differential Abilities of Agonists in Promoting Receptor Internalization in Brain

Here we showed that U50,488H, MOM-SalB, and 42B promoted KOR internalization in the VTA, but nalfurafine did not. Differential internalization by agonists has been observed on the DOR (Pradhan et al., 2009; Pradhan et al., 2010). Both selective DOR agonists SNC80 and AR-M100390 reduced complete Freud’s adjuvant-induced inflammatory pain. Pretreatment of mice with SNC80 abolished the subsequent responses to either agonist and promoted DOR internalization in mice expressing DOR conjugated with eGFP. In contrast, AR-M100390 treatment did not affect the analgesic response to the subsequent agonist administration, nor did it cause DOR internalization. Thus, DOR internalization is related to tolerance. U50,488H at 5 mg/kg promoted robust KOR phosphorylation and internalization in mouse brains; however, repeated treatment with U50,488H (5 mg/kg, s.c. twice/day for 5 days) did not induce tolerance (Huang et al., 2022). Only when a very high dose of U50,488H (80 mg/kg, twice a day for days 1 and 2, once a day for day 3) was used was U50,488H tolerance in mice observed (Huang et al., 2022). McLaughlin et al. (2004) used an escalating dosing regimen (up to 75 mg/kg) to induce U50,488H tolerance and found that prolonged KOR phosphorylation contributed to KOR tolerance. Thus, short-term KOR phosphorylation and internalization induced by U50,488H in the analgesic dose range is not related to tolerance.

## Conclusion

Based on the results obtained with four KOR agonists (U50,488H, MOM-SalB, nalfurafine, and 42B), we conclude that KOR agonist-promoted analgesic and antiscratch effects and CPA are unrelated to KOR phosphorylation and internalization in male mice. Among the four agonists, U50,488H, MOM-SalB, and 42B produced hypolocomotion and KOR phosphorylation and internalization, but nalfurafine did not; however, there is no causal relationship. Mechanisms underlying the observations remain to be determined. It may be that each agonist promotes a different population of conformational states, which activate different downstream signaling pathways.

## Data Availability

The original contributions presented in the study are included in the article/Supplementary Material. Further inquiries can be directed to the corresponding author.
