# Supplementary material for: Agonist-Promoted Phosphorylation and Internalization of the Kappa Opioid Receptor in Mouse Brains: Lack of Connection With Conditioned Place Aversion
Source: Front Pharmacol. 2022 May 16;13:835809. doi: 10.3389/fphar.2022.835809 (PMC9149264; doi:10.3389/fphar.2022.835809)
Supplement: Supplementary file 1 [file DataSheet1.PDF]

Supplementary Materials

The majority of the data presented here were published previously by our lab (Wang et al., 2008;Liu et al., 2019;Cao et al., 2020). We compiled these data as Supplementary Materials to enhance readability of the manuscript.
Additional experiments were performed to examine effects of MOM-SalB on novelty-induced hyperlocomotion (Fig. S4) and effects of MOM-SalB and 2 mg/kg U50,488H on rotarod performance (Fig. S5).

Table S1. EC_{50} values and maximal effects of nalfurafine and 42B in stimulating $[^{35}S]GTP\gamma S$ binding to membranes of CHO cells stably expressing the MOR, DOR, KOR or NOR. DAMGO, DPDPE, (-)U50,488H, and N/OFQ were used as the reference full agonists for the MOR, DOR, KOR and NOR, respectively (Cao et al., 2020). Data of MOM-SalB activities at the KOR are included (Wang et al., 2008). Mean ± SEM (n=3-4)

	MOR		DOR		KOR		NOR		KOR/MOR selectivity
	EC_{50} (nM)	Max. Effect (%)	EC_{50} (nM)	Max. Effect (%)	EC_{50} (nM)	Max. Effect (%)	EC_{50} (nM)	Max. Effect (%)	
(-)U50,488H					5.12±0.37	99.65±1.24			
MOM-SalB					0.6±0.2	97.8±6.4			
Nalfurafine	3.11± 0.63	73.88±2.93	24.22±2.56	129.1± 3.2	0.097±0.018	90.90± 3.25	279.7±17.7	119.7±2.2	32.1
42b	214.9±50.4	48.87±2.45	334.5±35.1	134.7± 3.4	25.56±1.50	91.34± 1.15	1760±284	110.9±7.4	8.4
DAMGO	6.30± 0.43	99.42±1.07							
DPDPE			7.06± 0.76	97.67±1.92					
Nociceptin / Orphanin FQ							0.046±0.007	110.8±2.8	

Analgesic effects of U50,488H, MOM-SalB, nalfurafine and 42B in the formalin test

Each of the four agonists reduced time spent licking from 15 to 35 min following formalin injection (phase II reaction) in a dose-dependent manner, indicating analgesic effects (Liu et al., 2019;Cao et al., 2020). A_{50} values of the agonists were calculated and are shown in the figures.

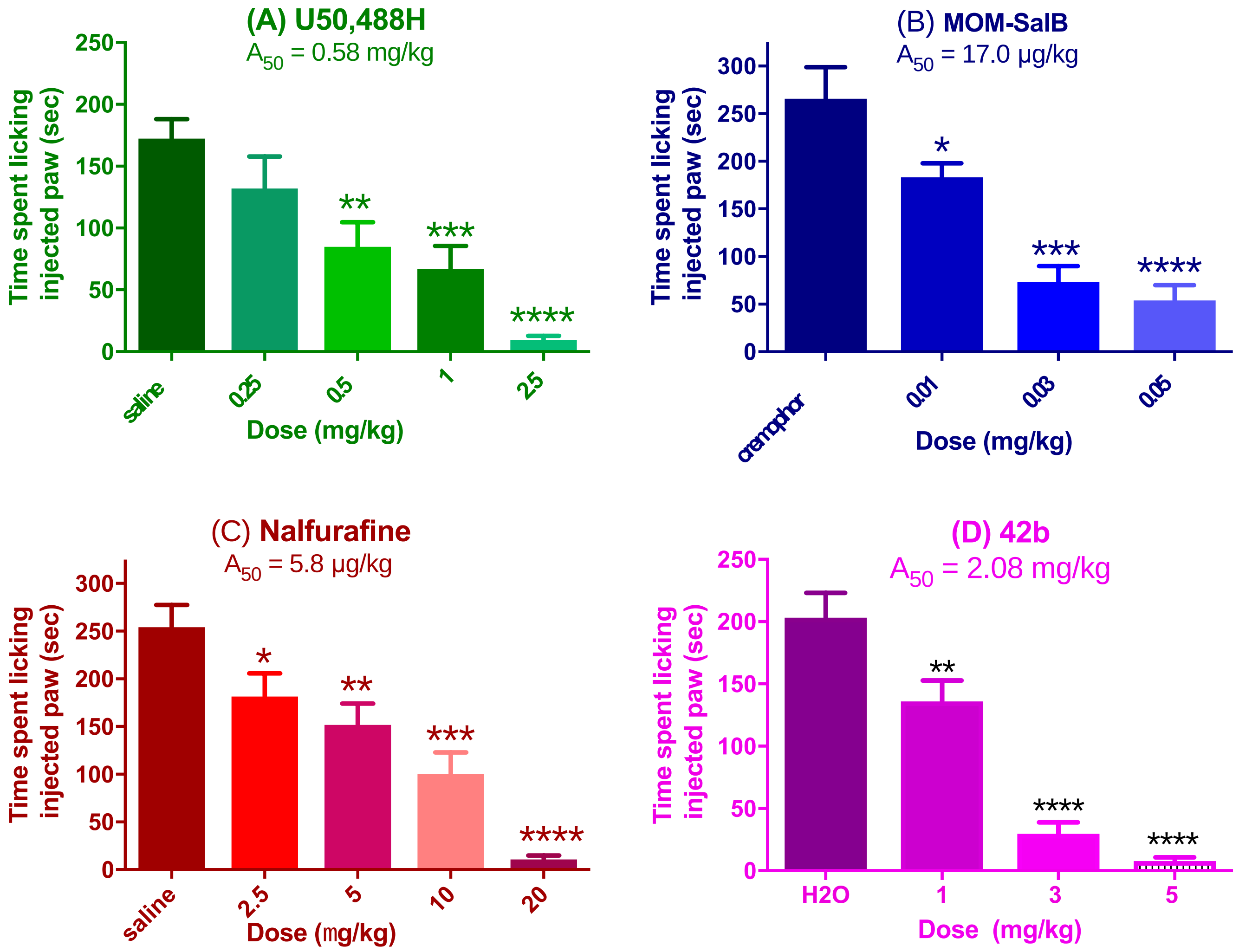

Figure S1. KOR agonists inhibited formalin-induced pain behaviors Data on U50,488H, MOM-SalB and nalfurafine are from Liu et al. (2019) and data on 42b are from Cao et al. (2020).
Mice were injected (s.c.) with vehicle or one dose of U50,488H, MOM-SalB, nalfurafine or 42b 5 min before injection of formalin into the right hind paws. The amount of time each animal spent licking the injected paw was counted for 20 min starting 15 min after formalin injection. A_{50} doses were determined. Data were analyzed using one-way ANOVA followed by Dunnett's *post-hoc* test. Significance levels are * $p < 0.05$, ** $p < 0.01$, *** $p < 0.001$ and **** $p < 0.0001$, compared to vehicle control, from Dunnett's *post hoc* test (mean ± SEM, n= 6-10 animals/group).

Inhibition of scratching induced by compound 48/80 by U50,488H, MOM-SalB, nalfurafine and 42B

Each of the four agonists reduced the number of scratching bouts induced by compound 48/80 in a dose-dependent manner (Liu et al., 2019;Cao et al., 2020). A_{50} values of the agonists were calculated and are shown in the figures.

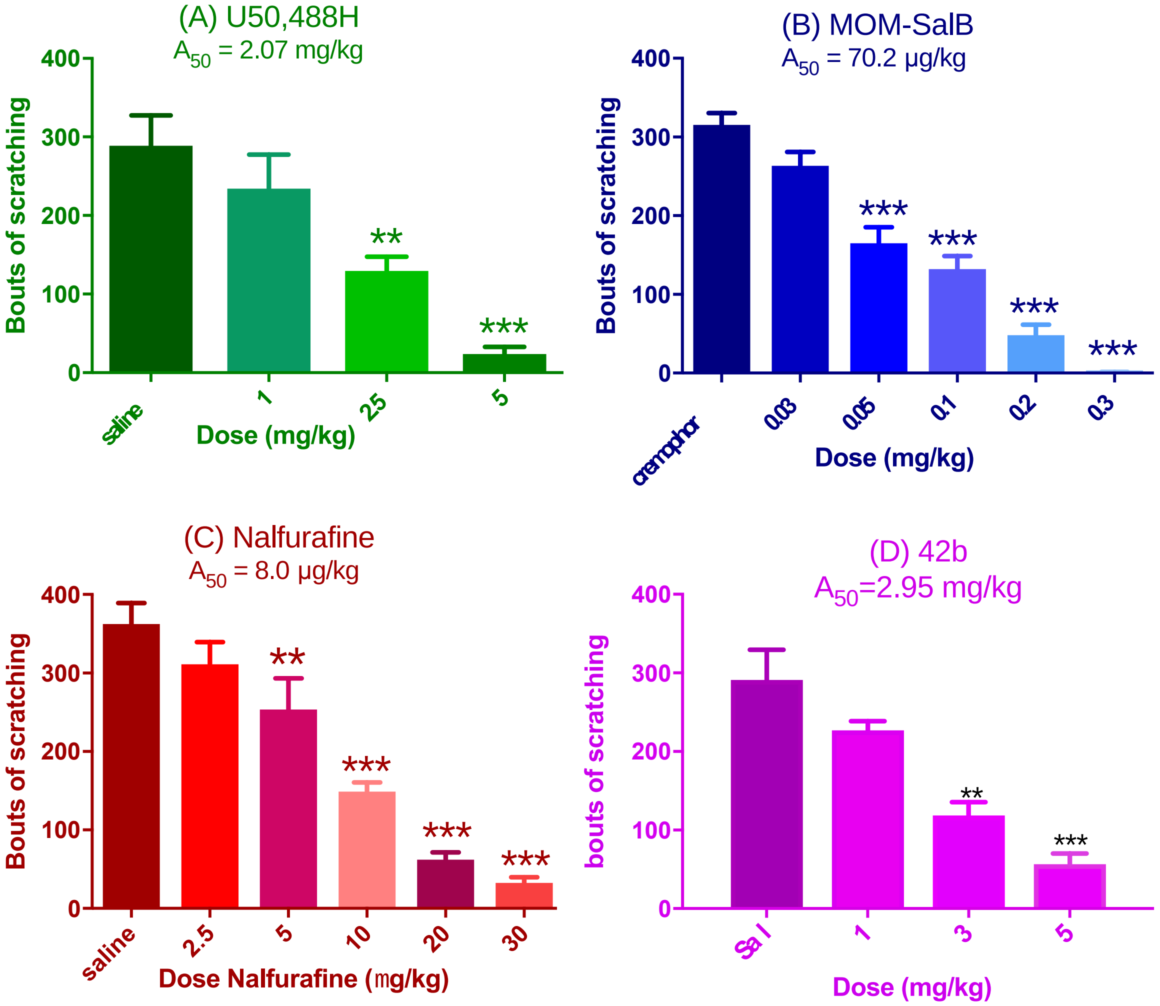

Figure S2. KOR agonists inhibited scratching behavior induced by compound 48/80. Data on U50,488H, MOM-SalB and nalfurafine are from Liu et al. (2019) and data on 42b are from Cao et al. (2020).
Mice were injected (s.c.) with vehicle or one of the different doses of U50,488H, MOM-SalB, nalfurafine or 42b followed by 20 min later with compound 48/80 into the nape and the bouts of scratching were counted for 30 min. A_{50} doses were determined. Data were analyzed using one-way ANOVA followed by Dunnett's *post-hoc* test. Significance levels are ** $p < 0.01$, *** $p < 0.001$, compared to vehicle control by Dunnett's *post hoc* test (mean ± SEM, n= 6-12 animals/group).

Effects of U50,488H, MOM-SalB, nalfurafine and 42B on CPA

U50,488H at 0.25, 0.5, 1, 2.5, 5 and 10 mg/kg or MOM-SalB at 0.03, 0.05, 0.1, 0.3 mg/kg caused profound CPA with no dose-dependent effects (Liu et al., 2019). In contrast, nalfurafine at 2.5, 5, 10 and 20 μg/kg or 42B at 1, 3 and 5 mg/kg did not (Liu et al., 2019;Cao et al., 2020).

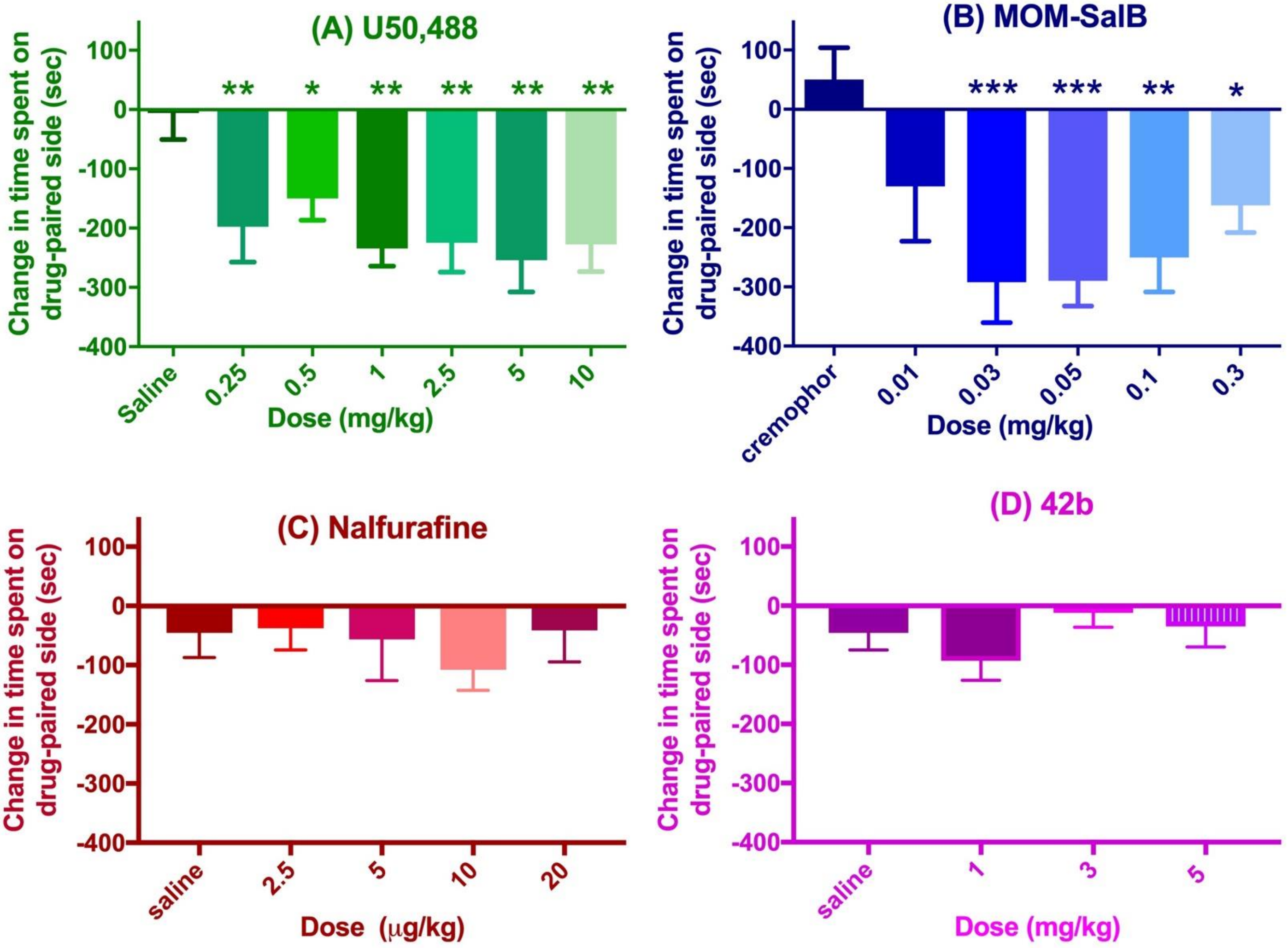

Figure S3. U50,488H (A) and MOM-SalB (B) caused CPA, whereas nalfurafine (C) and 42b (D) did not. Data on U50,488H, MOM-SalB and nalfurafine were from Liu et al. (2019). Data on 42b were from Cao et al. (2020).

On Day 0 (pre-test), mice were tested for preference for either side of the 2-chamber boxes. On Days 1-6, mice were injected with vehicle or one of the doses of U50,488H, MOM-SalB or nalfurafine 15 min before each 30-min conditioning session (2 sessions/day) for 6 days. On Day 7 (post-test), the time the animal spent on the drug-paired side was measured. The preference score was calculated as the length of time animal spent during the post-test subtracting the amount of time spent during the pre-test. Significance levels are * $p < 0.05$, ** $p < 0.01$, *** $p < 0.001$ compared to vehicle control (mean ± SEM, n= 8-10 animals/group) by Dunnett's *post hoc* test.

Effects of U50,488H, MOM-SalB, nalfurafine and 42B on locomotor activity

U50,488H at 5 mg/kg or 42B at 5 mg/kg significantly decreased ambulatory activities, but nalfurafine at 20 μg/kg did not have significant effects, compared with the control (Liu et al., 2019;Cao et al., 2020). MOM-SalB at 200 μg/kg caused significant inhibition of ambulatory activities.

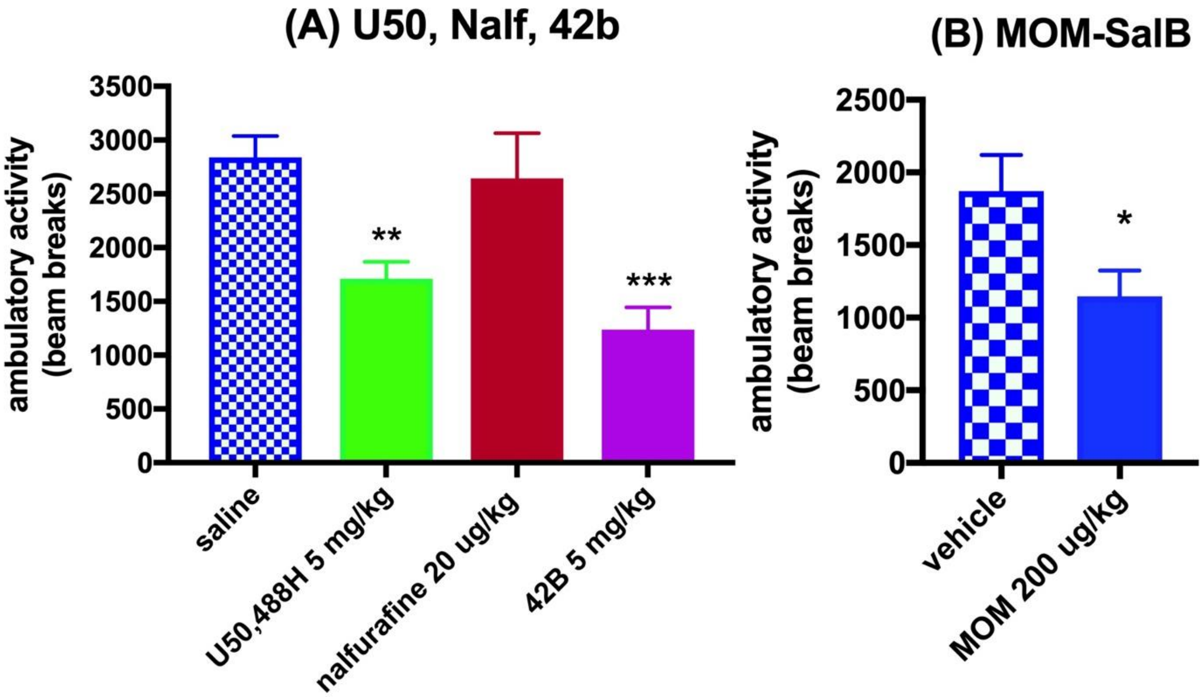

Figure S4. U50,488H, MOM-SalB, and 42b reduced novelty-induced hyperlocomotion, but nalfurafine did not. (A) U50,488H and nalfurafine data were from Liu et al. (2019). Data on 42b were from Cao et al. (2020). Mice were treated s.c. with (A) saline, 42B (5 mg/kg), U50,488H (5 mg/kg) or nalfurafine (20 μg/kg). **(B)** Mice were injected with vehicle (ethanol: Kolliphor: water 1:1:98) or MOM-SalB (200 μg/kg, s.c.). Locomotor activities were monitored and cumulative data between 0-30 min post-injection are shown here. Each value represents mean ± SEM (n = 8-14). ** $p<0.01$, *** $p<0.001$ compared to saline, by one-way ANOVA followed by Dunnett's multiple comparison's test n=11 (saline), 10 (U50) and 8 (nalf), 8(42b). **(B)** *$p<0.05$, compared to vehicle [ethanol: Kolliphor EL: water (1:1:98)] by two-tailed *t* test, n=13 each.

Effects of U50,488H, MOM-SalB and nalfurafine in the rotarod test

U50,488H reduced time spent on the rod in dose-dependent manner (Fig. S5A). At 2 mg/kg, U50,488H had significant effects at 30- and 40-min post infection. At 5 mg/kg, U50,488H caused significant reduction in the time staying on the rod at 10, 20, 30 and 40 min after injection (Liu et al., 2019).

Nalfurafine at 20 μg/kg caused significant motor incoordination at 30 and 40 min (Liu et al., 2019) (Fig. S5A), but to much lower extents than U50,488H at 5 mg/kg or MOM-SalB at 200 μg/kg.

MOM-SalB at 200 μg/kg impaired performance profoundly in the rotarod test at 10, 20, 30 and 40 min after injection; however, 70 μg/kg MOM-SalB impaired performance only at 10 min (Fig. S5B).

42B at 1, 3 or 5 mg/kg produced dose-dependent impairment in rotarod performance and at 5 mg/kg impaired the performance at 10, 20, 30 and 40 min and at 1 or 3 mg/kg 42b reduced time on the rod at 10, 20 and 30 min (Cao et al., 2020) (Fig. S5C).

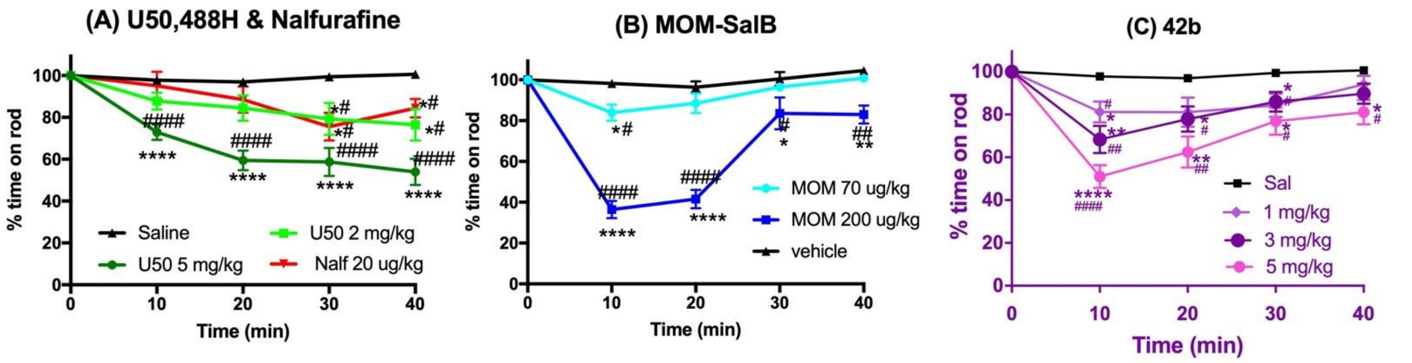

Figure S5. While (A) U50,488H (2 and 5 mg/kg), (B) MOM-SalB (70 and 200 μg/kg), and (C) 42b (1, 3, 5 mg/kg) impaired rotarod performance, (A) nalfurafine (20 μg/kg) had only a minor effect Data on U50,488H (5 mg/kg) and nalfurafine are from Liu et al. (2019) and Data on 42b are from Cao et al. (2020). After training on the previous day, mice were injected s.c. with **(A and C)** saline, U50,488H (2, 5 mg/kg), 42B (1, 3, 5 mg/kg) or nalfurafine (20 μg/kg) or with (B) vehicle (ethanol: Kolliphor: water 1:1:98) or MOM-SalB (70 or 200 μg/kg) and tested on the rotarods 10, 20, 30, and 40 min after injection. The time each stayed on the rods was recorded and normalized against the baseline. Data were analyzed with two-way ANOVA followed by Dunnett's multiple comparisons test. *p<0.05, ** p<0.01, ***p<0.001, ****p<0.0001, compared to Time 0 of the same treatment group. # p<0.05, ## p<0.01, ###p<0.001, ####p<0.0001, compared to vehicle at the same time point. Each value is mean ± SEM (n= 8-12/group)

References

Cao, D., Huang, P., Chiu, Y.T., Chen, C., Wang, H., Li, M., Zheng, Y., Ehlert, F.J., Zhang, Y., and Liu-Chen, L.Y. (2020). Comparison of Pharmacological Properties between the Kappa Opioid Receptor Agonist Nalfurafine and 42B, Its 3-Dehydroxy Analogue: Disconnect between in Vitro Agonist Bias and in Vivo Pharmacological Effects. *ACS Chem Neurosci* 11**,** 3036-3050.

Liu, J.J., Chiu, Y.T., Dimattio, K.M., Chen, C., Huang, P., Gentile, T.A., Muschamp, J.W., Cowan, A., Mann, M., and Liu-Chen, L.Y. (2019). Phosphoproteomic approach for agonist-specific signaling in mouse brains: mTOR pathway is involved in kappa opioid aversion. *Neuropsychopharmacology* 44**,** 939-949.

Wang, Y.L., Chen, Y., Xu, W., Lee, D.Y.W., Ma, Z.Z., Rawls, S.M., Cowan, A., and Liu-Chen, L.Y. (2008). 2-methoxymethyl-salvinorin B is a potent kappa opioid receptor agonist with longer lasting action in vivo than salvinorin A. *J. Pharmacol. Exp. Ther.* 324**,** 1073-1083.
